# Ocrelizumab Discontinuation After Recurrent Serious Infections in Clinically Stable Primary Progressive Multiple Sclerosis: A Case Report

**DOI:** 10.7759/cureus.116594

**Published:** 2026-09-20

**Authors:** Tyler Lo, Lamisa Hassan, Shane Wilson, Rithvik Seri, Stephanie Hang

**Affiliations:** 1 Medicine, Touro College of Osteopathic Medicine, Montana, Great Falls, USA; 2 Internal Medicine, Benefis Health System, Great Falls, USA

**Keywords:** anti-cd20 therapy, ocrelizumab, primary progressive multiple sclerosis, serious infection, shared decision-making, treatment de-escalation

## Abstract

Long-term anti-CD20 therapy in primary progressive multiple sclerosis (PPMS) requires balancing expected neurologic benefit against infection risk as patients age and accumulate disability. A 61-year-old woman with PPMS and no recent clinical or radiographic evidence of inflammatory disease activity while receiving ocrelizumab every six months was hospitalized three times over approximately five months. The first hospitalization was complicated by viridans group streptococcal bacteremia associated with sialadenitis and a progressive pulmonary infiltrate with acute hypoxemic respiratory failure. She was readmitted with multifocal pneumonia, recurrent hypoxemia, metabolic encephalopathy, dysphagia with suspected aspiration, malnutrition, and further functional decline. She was later hospitalized a third time for a stage IV sacral pressure injury complicated by a methicillin-resistant *Staphylococcus aureus* (MRSA) abscess and sacral osteomyelitis. Serial neurologic evaluations showed no evidence of relapse, and magnetic resonance imaging revealed no active demyelination throughout. Although ocrelizumab may have increased susceptibility to infection, multiple coexisting risk factors, including aspiration risk, frailty, immobility, malnutrition, pressure injury, and recent healthcare exposure, complicated causal attribution. After the first two infectious admissions, the next scheduled infusion was withheld; following the osteomyelitis admission, neurology recommended discontinuation, and the patient remained off disease-modifying therapy without documented relapse at the last follow-up. This case underscores the need to reassess long-term therapy according to recent disease activity, cumulative treatment exposure, immunologic monitoring, infection history, functional status, comorbidities, care context, and patient goals. A single case cannot establish age-based or universal discontinuation thresholds; instead, it supports individualized shared decision-making regarding treatment continuation, interval extension, temporary interruption, or discontinuation.

## Introduction

Multiple sclerosis (MS) is a chronic immune-mediated demyelinating disease of the central nervous system and a leading cause of nontraumatic neurologic disability in young adults [1]. Its pathogenesis remains incompletely understood but involves interactions between adaptive and innate immune mechanisms, leading to central nervous system inflammation, demyelination, and progressive axonal loss [1]. Over the past two decades, disease-modifying therapies (DMTs) have substantially reduced inflammatory disease activity and delayed disability accumulation, particularly in relapsing forms of MS [2]. However, ongoing treatment with many DMTs entails a trade-off between disease control and treatment-related adverse effects, including infection. Anti-CD20 therapies demonstrate this trade-off: prolonged B-cell depletion can impair humoral immune responses and increase the risk of serious infection, particularly in older patients and those with advanced disability [3,4].

As people with MS age and accumulate longer treatment exposure, clinicians must determine whether continued DMT provides net clinical benefit [5]. This question is particularly relevant in progressive MS without recent inflammatory activity, in which the expected benefit of continued therapy may diminish while age, disability, frailty, recurrent infections, and comorbidities increase treatment-related risk [4,5]. No universally accepted criteria define when DMT should be de-escalated or discontinued in MS, leaving clinicians with limited guidance when treatment-related risk begins to approach anticipated benefit [6,7]. Consequently, these decisions often depend on individualized clinical judgement and shared decision-making.

We present a patient with primary progressive multiple sclerosis (PPMS) receiving ocrelizumab who developed recurrent severe infections and respiratory failure, raising concern about the safety of continued therapy. Rather than proposing a universal threshold, this case illustrates how disease-related factors, patient characteristics, and clinical context can be integrated into individualized decisions about treatment continuation or de-escalation.

## Case presentation

First admission (day 0)

A 61-year-old woman with fibromyalgia and PPMS, diagnosed in 2002, presented to the emergency department on day 0 with acute altered mental status characterized by disorientation, confusion, noncommunicative behavior, and inability to recognize family members. Her husband reported no clinical relapses or new radiographic findings in the preceding years. She had received DMT for many years, initially glatiramer acetate (Copaxone) by subcutaneous injection three times weekly, subsequently transitioned to ocrelizumab (Ocrevus) at six-month intervals, with her most recent infusion approximately two months before presentation (day -60).

**Figure 1:**
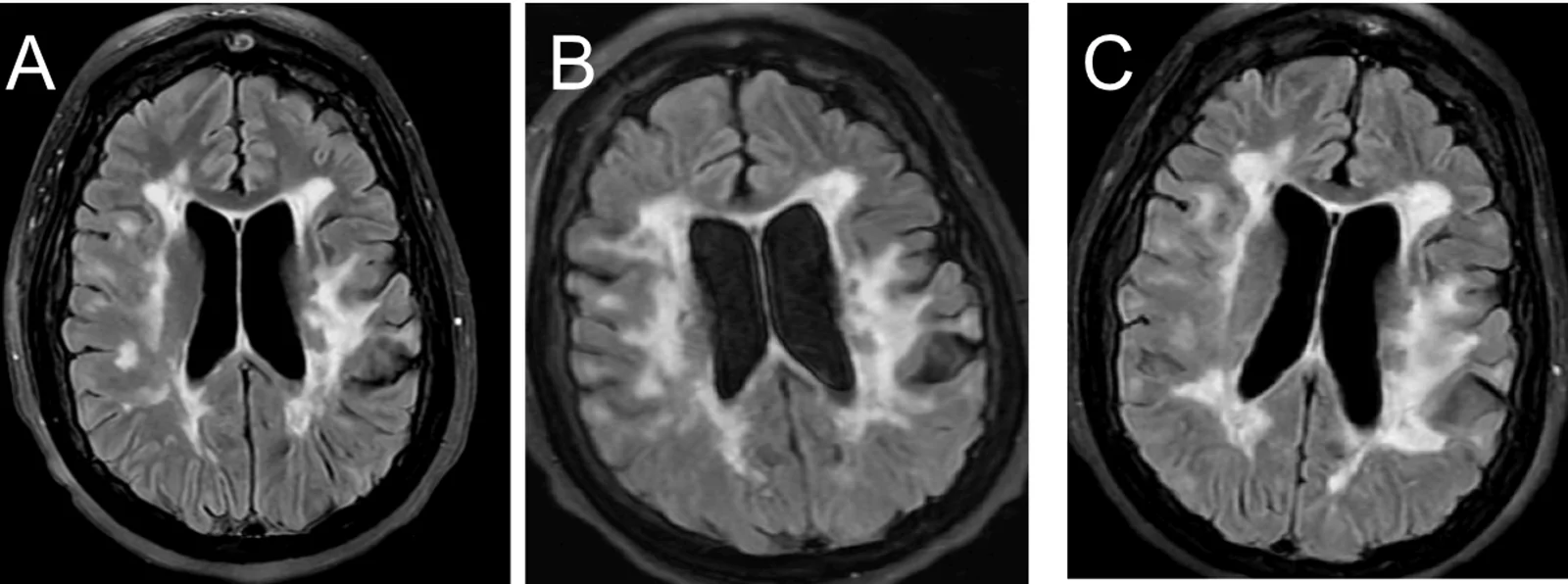
Representative axial T2-FLAIR brain MRI at three timepoints: (A) ~4 years before admission 1, (B) day 1, and (C) day 53. The distribution of confluent supratentorial periventricular and subcortical T2-FLAIR hyperintensities remained similar across examinations. The corresponding radiology reports documented no abnormal postcontrast enhancement or diffusion restriction, with no radiographic evidence of active demyelination. FLAIR: fluid-attenuated inversion recovery

Laboratory evaluation demonstrated leukocytosis with marked lymphopenia (Table 1). Noncontrast head CT and CT angiography of the head and neck showed no acute intracranial abnormality, vascular occlusion, or high-grade stenosis but revealed incidental right parotid and submandibular sialadenitis with adjacent reactive lymphadenopathy. Compared with prior imaging, brain MRI demonstrated stable supratentorial and infratentorial demyelinating plaques without enhancement or other evidence of active demyelination (Figure 1A-1B). Electroencephalogram (EEG) showed no electrographic seizures. Cerebrospinal fluid (CSF) evaluation showed no pleocytosis, mild protein elevation, no growth on culture, and negative meningitis and encephalitis testing (Table 2).

**Table 1:** Selected laboratory findings over time. Day 0 represents the first hospital admission. Quantitative immunoglobulins and CD19+ B-cell counts were not obtained during any admission; their absence is addressed in the Limitations section. H: high; L: low; WBC: white blood cell count; ANC: absolute neutrophil count; ALC: absolute lymphocyte count

Parameters	Patient values			Reference range
	Day -406	Day 0	Day 53	
WBC, ×10³/µL	11.7	18.0 H	14.8 H	3.8-11.8
ANC, ×10³/µL	8.90 H	17.18 H	13.72 H	1.90-8.20
ALC, ×10³/µL	1.43	0.30 L	0.46 L	1.10-3.10
Albumin, g/dL	4.6	4.1	2.5 L	3.5-5.7

**Table 2:** Neurologic, radiographic, and CSF findings across serial evaluations. Day 0 represents the first hospital admission. Baseline imaging was obtained approximately four years before the first admission. MRI findings remained stable without radiographic evidence of active demyelination across serial examinations. CSF: cerebrospinal fluid; EEG, electroencephalogram; WBC: white blood cell count; H: high; NA: not available/not obtained

Parameters	Patient values					Reference range
	Baseline	Day 0	Day 1	Day 53	Day 56	
Brain MRI	Stable plaques	NA	Stable; no active demyelination	Stable; no enhancement or diffusion restriction	NA	-
Spine MRI	NA	NA	NA	Chronic MS lesions; no acute findings	NA	-
CSF WBC, cells/µL	NA	4	NA	NA	1	0-5
CSF protein, mg/dL	NA	60 H	NA	NA	34	15-45
CSF glucose, mg/dL	NA	105 H	NA	NA	59	40-70
CSF culture	NA	No growth	NA	NA	NA	-
Meningitis panel	NA	Not detected	NA	NA	Not detected	-

During admission, she developed viridans group streptococcal bacteremia (*Streptococcus intermedius*) attributed to the right parotid sialadenitis and was treated with ceftriaxone and metronidazole. She subsequently developed a progressive left lower lobe infiltrate with acute hypoxemic respiratory failure requiring supplemental oxygen. By day 7, her mentation had substantially improved, with residual mild confusion and no focal deficits, and she was transferred to inpatient rehabilitation with baseline functional deficits attributable to her MS. C-reactive protein (CRP) remained markedly elevated at 154.9 mg/L on day 22.

Second admission (day 38)

Shortly after discharge, the patient was transferred from a transitional care facility back to the emergency department on day 38 with progressive cough, congestion, hypoxia, generalized weakness, and worsening confusion. Chest imaging revealed bilateral scattered ground-glass opacities and lower lobe consolidations consistent with multifocal pneumonia, with subsequent worsening. A respiratory panel, urinary antigens for *Streptococcus pneumoniae* and *Legionella pneumophila*, and peripheral blood cultures were negative. She received sequential broad-spectrum antibiotics, including cefepime, vancomycin, and metronidazole.

Given suspected oropharyngeal dysphagia and concern for aspiration, pulmonology and infectious disease recommended completing the antibiotic course with pulmonary hygiene and dietary modification rather than escalating antimicrobial therapy. She required 4-5 L/min oxygen by nasal cannula, weaned as tolerated. Fluctuating mental status was attributed to metabolic encephalopathy. Repeat brain MRI on day 53 remained similar to prior examinations without enhancement or diffusion restriction, providing no radiographic evidence of active demyelination (Figure 1C). Whole-spine MRI, repeat EEG, and repeat CSF evaluation likewise showed no acute neurologic abnormality or evidence of central nervous system infection (Table 2). Neurology therefore recommended supportive management.

She developed a stage III sacral pressure injury, managed conservatively, and experienced a 4.3% reduction in body weight. CRP had decreased to 39.5 mg/L by day 69. She was discharged on day 76, afebrile and hemodynamically stable, requiring 4-5 L/min oxygen for transfer to the transitional care unit. Across both admissions, there was no clinical or radiographic evidence of MS activity.

Third admission (day ~140)

Approximately 4.5 months after presentation (day ~140), the patient was hospitalized for the progression of her sacral pressure injury to a stage IV ulcer complicated by abscess and osteomyelitis. MRI demonstrated a 3-cm abscess with fistulous communication to the skin and findings consistent with sacral osteomyelitis. CRP had further declined to 12.1 mg/L by day 160. She underwent excisional debridement extending to the fascia and bone with sacral bone biopsy and negative-pressure wound therapy; intraoperative cultures grew methicillin-resistant *Staphylococcus aureus* (MRSA). Infectious disease recommended a six-week course of intravenous antimicrobial therapy coordinated with outpatient parenteral antimicrobial therapy and peripherally inserted central catheter (PICC) placement.

She remained afebrile and hemodynamically stable with mild anemia and preserved renal function and had substantial baseline functional dependence requiring maximal assistance for mobility and activities of daily living. Given her recurrent serious infections, neurology recommended discontinuing ocrelizumab.

Neurologic management and follow-up

Inpatient neurologic evaluations across all admissions demonstrated no clinical or radiographic evidence of MS exacerbation. Following the first two infectious admissions, the next scheduled ocrelizumab infusion was withheld, and no alternative DMT was initiated, with long-term management deferred to outpatient neurology. After the subsequent hospitalization for MRSA-associated sacral osteomyelitis, neurology recommended discontinuation. At the most recent available follow-up, the patient remained off long-term DMT without documented clinical relapse, though with ongoing infectious morbidity related to the pressure ulcer and osteomyelitis.

## Discussion

MS and DMT

MS is a chronic immune-mediated disorder of the central nervous system driven by genetic, environmental, and immune factors, with pathogenic lymphocyte trafficking across the blood-brain barrier contributing to demyelination and neurodegeneration [8-10]. MS includes relapsing-remitting, primary progressive, and secondary progressive phenotypes; PPMS accounts for approximately 10-15% of cases and is characterized by gradual neurologic decline without distinct relapses or remissions [11,12]. Because this patient received long-term ocrelizumab for PPMS, the following discussion focuses on the benefits and risks of anti-CD20 therapy.

Anti-CD20 therapy: benefit versus risk

MS treatment has shifted toward high-efficacy DMTs, most notably anti-CD20 monoclonal antibodies. Ocrelizumab was the first and remains the only DMT approved for PPMS, reducing confirmed disability progression, slowing brain volume loss, and preserving upper-extremity function in this population. By depleting CD20+ B lymphocytes, ocrelizumab reduces antigen presentation, pro-inflammatory cytokine production, and immune activation central to MS pathogenesis. Recent randomized data indicate that this benefit is not confined to younger patients. The phase 3b ORATORIO-HAND trial, which enrolled older and more disabled patients, demonstrated that ocrelizumab reduced disability progression with effects extending to patients up to age 65 and to those older than 55 with more advanced disability, cautioning that rigid age-based cutoffs may lack biological justification [13]. Continued therapeutic benefit is therefore plausible even in older PPMS patients and should not be assumed to be absent.

The potential benefits of ocrelizumab must be weighed against the risks of sustained B-cell depletion. Anti-CD20 treatment can impair humoral immunity, reduce vaccine responses, and contribute to hypogammaglobulinemia, particularly after prolonged exposure [14]. Observational studies have associated infection or hypogammaglobulinemia with factors such as older age, longer treatment duration, lower baseline IgG, lymphopenia, prior DMT exposure, and progressive MS, although findings have not been uniform across cohorts [3,4,15,16]. 

The prescribing information recommends monitoring quantitative serum immunoglobulins during treatment and after discontinuation until B-cell repletion, particularly after recurrent serious infections, and considering discontinuation in patients with recurrent or opportunistic serious infections or prolonged hypogammaglobulinemia requiring immunoglobulin replacement [17]. 

This patient's infectious burden exceeded what is typically reported in the pivotal trials. In ORATORIO-HAND, serious infections occurred in 6% of ocrelizumab-treated patients (2% when COVID-19 was excluded) versus 5% with placebo, were predominantly respiratory and urinary tract infections, and included no serious opportunistic infections [13]. Even in the older (>55 years) and more disabled subgroups most relevant to this patient, serious infections reached only approximately 9% [13]. Pooled all-exposure safety data demonstrated a similar serious infection rate at roughly 2.0 serious infections per 100 patient-years, most commonly urinary tract infection and pneumonia [3]. By contrast, this patient developed viridans group streptococcal bacteremia, multifocal pneumonia with hypoxemic respiratory failure, and MRSA sacral osteomyelitis requiring operative debridement and six weeks of intravenous antimicrobials, an infectious burden both more frequent and more severe than characteristic of these trial populations. 

Importantly, infection risk in advanced PPMS cannot be attributed to immunotherapy alone. As patients age and accumulate disability, secondary complications such as dysphagia with impaired airway protection, neurogenic bladder, malnutrition, pressure injury, and repeated hospitalization can independently increase the risk of aspiration pneumonia, urinary tract infection, skin and soft-tissue infection, and healthcare-associated infection [4,5]. When these vulnerabilities compound age-related immunosenescence and prolonged B-cell depletion, the cumulative risk of serious infection can rise substantially even as measurable inflammatory disease activity wanes [4,5]. Management of advanced PPMS therefore requires balancing the benefits of continued disease control against an evolving infection risk defined by each patient's functional status, comorbidity burden, and overall trajectory. 

Clinical relevance and proposed de-escalation considerations 

In this patient, reassessment of ocrelizumab required the consideration of three overlapping domains: recent MS activity, individual vulnerability to infection, and the broader clinical context. These factors do not establish that ocrelizumab caused the recurrent infections; rather, they illustrate how the balance between expected neurologic benefit and treatment-related risk may shift as progressive disease, disability, and recurrent infection accumulate.

Disease-Related Factors

The patient had no recent clinical relapse and no evidence of active demyelination on serial MRI. Inflammatory disease activity in PPMS generally decreases with advancing age and disease duration, which may reduce the relative benefit of continued anti-inflammatory therapy [5]. However, randomized and real-world evidence suggests that ocrelizumab may continue to provide benefit in some older or more disabled patients [13,18]. Her clinical course therefore suggested a potentially lower, but not absent, expected benefit from continued treatment.

Patient-Related Factors

At 61 years of age, the patient had several features associated with increased vulnerability to infection, including progressive functional impairment, dysphagia with suspected aspiration, immobility, nutritional decline, and pressure injury. Her laboratory course exhibited sustained lymphopenia during both acute admissions (absolute lymphocyte count 0.30 and 0.46×10³/µL), consistent with the treatment-associated lymphopenia linked to infection risk in observational cohorts [4,15,16]. Her first hospitalization included culture-confirmed viridans group streptococcal bacteremia associated with sialadenitis and subsequent pulmonary infection, while the second was complicated by multifocal pneumonia, suspected aspiration, and hypoxemic respiratory failure. Her subsequent hospitalization for a stage IV pressure ulcer complicated by MRSA abscess and sacral osteomyelitis further demonstrated persistent infectious vulnerability. Although prolonged B-cell depletion may have contributed to infection susceptibility, the recurrent infections occurred in the setting of multiple competing risks, including aspiration, immobility, pressure injury, nutritional decline, and repeated healthcare exposure.

Clinical Context

Repeated hospitalization, transitional care placement, broad-spectrum antimicrobial exposure, and progressive deconditioning further increased her vulnerability to subsequent complications. These circumstances were not direct manifestations of MS but became clinically relevant to the decision regarding continued immunotherapy. The case therefore illustrates why treatment reassessment in advanced PPMS cannot rely solely on age, imaging stability, or infection history in isolation.

Following the first two infectious hospitalizations, the patient's next scheduled ocrelizumab infusion was withheld, and no alternative DMT was initiated. With no evidence of acute inflammatory disease activity, long-term management was initially deferred to outpatient neurology. After a subsequent hospitalization for MRSA-associated sacral osteomyelitis, neurology recommended discontinuation of ocrelizumab. At the most recent follow-up, she remained off DMT without documented clinical relapse. This course highlights how persistent infectious morbidity can shift the benefit-risk balance even when PPMS remains clinically stable.

Evidence regarding DMT discontinuation in older patients remains mixed. In DISCOMS, patients older than 55 years with prolonged clinical and radiographic stability had low rates of new disease activity overall, although events were more frequent after discontinuation [19]. Similarly, a matched cohort of patients aged 50 years and older with nonactive MS found no statistically significant difference within the anti-CD20 subgroup after treatment discontinuation [20]. Neither study specifically evaluated patients with PPMS receiving long-term ocrelizumab after recurrent serious infections, limiting direct applicability to this case.

Management after recurrent serious infection may include continued dosing, temporary interruption, extended-interval dosing, or discontinuation based on recent disease activity, immune status, infection history, functional trajectory, and patient preferences [5-7]. Extended-interval dosing may offer an intermediate strategy, although PPMS-specific evidence remains limited. In this patient, recurrent infections initially prompted treatment interruption and ultimately led neurology to recommend discontinuation after sacral osteomyelitis.

**Figure 2:**
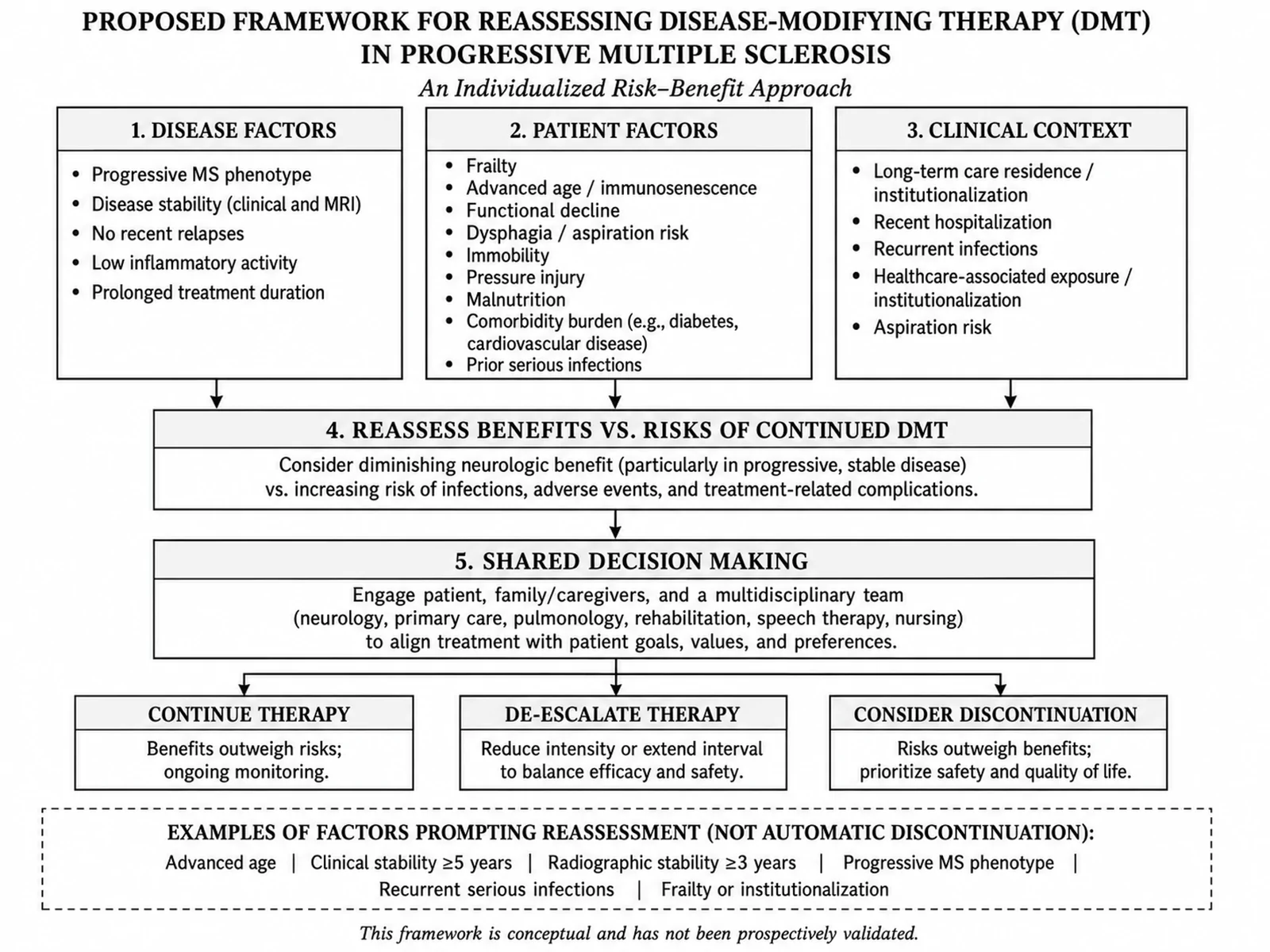
Disease-patient-clinical framework for disease-modifying therapy reassessment in progressive multiple sclerosis. This conceptual framework integrates disease-related factors, patient-specific vulnerability, and clinical context to support individualized reassessment of continued therapy, de-escalation, or discontinuation. The framework has not been prospectively validated. DMT: disease-modifying therapy; MS: multiple sclerosis Image Credits: Tyler Lo and Lamisa Hassan. Created using Microsoft PowerPoint (Microsoft Corporation, Redmond, WA, USA). Framework informed by references [5-7,13,17-20].

These considerations are summarized in the proposed disease-patient-clinical framework (Figure 2), which is intended to support structured reassessment of benefit versus risk and multidisciplinary shared decision-making regarding continuation, interval modification, temporary interruption, or discontinuation of DMT. The framework is conceptual and has not been prospectively validated.

Limitations

This report has several limitations. A single case cannot establish that ocrelizumab caused any of the infectious episodes. Infectious workup for the pneumonia was unrevealing, and aspiration was suspected rather than confirmed, and frailty, dysphagia, immobility, nutritional decline, pressure injury, and recent healthcare exposure were important competing factors. Quantitative immunoglobulin and CD19+ B-cell measurements were not obtained; their absence limits the direct assessment of treatment-related immune suppression, although the documented lymphopenia provides partial supporting evidence. 

## Conclusions

After recurrent serious infections culminating in MRSA-associated sacral osteomyelitis, ocrelizumab was withheld and subsequently recommended for discontinuation while the patient's PPMS remained clinically stable during available follow-up. This case does not establish that ocrelizumab caused the infections or define a universal stopping threshold. Rather, it illustrates how recent inflammatory disease activity, cumulative treatment exposure, infection history, functional status, aspiration risk, pressure injury, immune status, and patient goals should be considered together when reassessing long-term anti-CD20 therapy in PPMS.
